# Association between Corneal Higher-Order Aberrations Evaluated with a Videokeratographer and Corneal Surface Abnormalities in Dry Eye

**DOI:** 10.3390/diagnostics13213319

**Published:** 2023-10-26

**Authors:** Natsuki Kusada, Norihiko Yokoi, Chie Sotozono

**Affiliations:** Department of Ophthalmology, Kyoto Prefectural University of Medicine, Kyoto 602-8566, Japan; nkusada9@koto.kpu-m.ac.jp (N.K.); csotozon@koto.kpu-m.ac.jp (C.S.)

**Keywords:** dry eye disease, higher-order aberrations, visual function, videokeratography

## Abstract

Analysis of higher-order aberrations (HOAs) is one reported method for evaluating dry eye disease (DED)-related loss of visual function. Tear film (TF) instability and corneal epithelial damage (CED) are both reportedly responsible for HOAs in DED, although, to the best of our knowledge, there are no reported methods that allow concurrent evaluation of their effects. In this study, we used a videokeratographer (VK) to continuously measure HOAs in DED after eye opening and investigated factors of ocular surface abnormalities that determine HOAs. This study involved 96 DED cases that underwent DED symptom assessment with a questionnaire and examination of tear volume, TF abnormalities (i.e., TF lipid-layer interference grades and spreading grades, and non-invasive breakup time and fluorescein breakup time), and CED, and their correlation with HOAs evaluated via VK. The results show that HOAs at 1 or 2 s after eye opening can reflect TF instability and CED within the central 4-millimeter-diameter area of the optical zone in DED eyes concurrently. This finding may be useful for the rapid and non-invasive detection and evaluation of degraded visual function in DED cases with a variety of clinical features.

## 1. Introduction

Tear film (TF) is comprised of two layers, the lipid layer and the aqueous layer, and it is the first optical surface through which light is transmitted into the eye. Thus, TF plays an important role in maintaining the smoothness of the corneal surface structure and ensuring high optical quality [1]. Dry eye disease (DED) is a disease of increasing prevalence worldwide [2] that causes a wide variety of symptoms, including ocular discomfort and impaired visual function (VF). According to the Asia Dry Eye Society, DED is defined as a multifactorial disease characterized by TF instability that is potentially accompanied by ocular surface damage [3]. This suggests that in DED patients, the impaired VF is caused by both TF instability, which results in TF breakup after eye opening, and the resultant various types of corneal epithelial damage (CED), which can be time-dependent, complex, and difficult to evaluate. Accordingly, it is speculated that those abnormalities have a significant impact on DED-associated visual impairment, although the exact level of significance has yet to be fully elucidated.

Several methods have been reported for the evaluation of visual impairment in DED. Subjective evaluation methods include the measurement of contrast sensitivity [4,5,6,7] and functional visual acuity [8,9,10], while objective evaluation methods include corneal topography analysis [11,12], the measurement of higher-order aberrations (HOAs) via wavefront analysis [13,14,15,16,17], the measurement of ocular light scattering [18,19], and modulation transfer function using double-pass retinal images [20,21]. However, there have been few reports on the association between visual impairment evaluated by these methods and ocular surface abnormalities or subjective symptoms in DED [10,12,18,22,23,24].

Videokeratographers (VKs) continuously record images of placido rings projected onto the cornea (i.e., Meyer-ring [MR] images), and the analysis of MR images is reportedly useful for evaluating TF [25,26,27,28,29,30,31]. We previously investigated the relationship between quantitative indicators of disturbance values in MR images obtained via VK and corneal surface abnormalities in DED and showed that VK can be used to evaluate the corneal surface abnormalities in DED, comprising TF instability and CED [32]. Furthermore, since VK can also measure HOAs from MR images, those measurements may possibly allow for detailed tracking and evaluation of variable-degraded VF in DED.

The purpose of this present study was to investigate the effectiveness of continuous measurement of HOAs via VK in DED patients and assess the corneal surface abnormalities responsible for HOAs.

## 2. Materials and Methods

### 2.1. Study Participants

This prospective study involved 96 eyes (36 right eyes, 60 left eyes) of 96 DED patients [12 males and 84 females; mean age: 61.8 ± 15.9 (mean ± SD) years] who were followed at the Dry Eye Outpatient Clinic of the Kyoto Prefectural University of Medicine Hospital, Kyoto, Japan. All patients were diagnosed with DED based on the Japanese DED diagnostic criteria at the time of their first visit to our clinic, based on DED symptoms and fluorescein breakup time (BUT) (FBUT) being 5 s or less. In all patients, the data from the eye with the more severe subjective symptoms was used. However, if the severity of symptoms was the same in both eyes, the right-eye data was used. Contact lens users, patients with an eyelid disease such as blepharospasm, blepharoptosis, lagophthalmos, entropion, or ectropion, and patients with a history of ocular surgery (other than cataract surgery) within 3 months prior to the initiation of the study, including those performed for eyelid disorders, glaucoma, conjunctivocorneal diseases, and punctal occlusion, were excluded from the study. The protocols of this study were approved by the Institutional Review Board of Kyoto Prefectural University of Medicine, and in accordance with the tenets set forth in the Declaration of Helsinki, written informed consent was obtained from all patients prior to their involvement in the study.

### 2.2. Items for Examination

All patients were first evaluated for subjective symptoms using a questionnaire and then evaluated by examinations in the order of (1) tear volume assessment using a videomeniscometer, (2) TF assessment using a videointerferometer, (3) HOAs analysis using VK, and (4) slit-lamp observation of ocular surface abnormalities using fluorescein staining.

### 2.3. Evaluation of Subjective Symptoms

All patients were evaluated using the Dry Eye-Related Quality-of-Life Score (DEQS) questionnaire on 15 items related to specific DED symptoms (i.e., irritation, eye dryness, pain, eye fatigue, heavy eyelids, and redness) and their impact on their daily lives (i.e., difficulty in opening the eye, blurred vision, sensitivity to light, eye-related problems when reading, eye-related problems when watching television or looking at a computer monitor or cell phone screen, a feeling of distraction due to eye symptoms, eye symptoms affecting work, not feeling like going outside due to eye symptoms, and mental depression due to eye symptoms) [33]. Patients answered on a scale of 1 to 4 (with the larger number indicating a greater burden) the questions regarding the frequency and severity of each disability experienced, if they experienced that symptom within the week before their initial visit (e.g., if there were no applicable symptoms, the patient was instructed to answer 0 for both frequency and severity). Finally, the overall degree of quality-of-life disability was calculated as a summary score (score: 0–100) using the following formula: summary score = [(sum of the degree scores for all questions answered) × 25]/(total number of questions answered).

### 2.4. Evaluation of Tear Volume and TF Stability

First, to evaluate tear volume, a videomeniscometer was used to measure the tear meniscus radius (TMR; mm) at the center of the inferior tear meniscus, which is known to correlate with the tear volume on the ocular surface [34]. Second, to evaluate TF stability, a videointerferometer (DR-1; Kowa Co., Ltd., Tokyo, Japan) was used to measure TF lipid layer (TFLL) interference grade (IG) and spread grade (SG) (both graded 1–5, with 1 being the best) and non-invasive BUT (NIBUT; in seconds). IG is an index based on interference pattern and is known to reflect DED parameters including CED and TF BUT, and it is especially useful for evaluating aqueous deficient dry eye (ADDE), while SG reflects the ability of the TFLL to spread upwardly and is reflected by aqueous tear volume [35,36]. NIBUT was measured as the time after eye opening until the area with TF breakup appeared. If no TF breakup was observed for 10 s after eye opening, NIBUT was evaluated as 10 s; if the eye closed before TF breakup was observed, NIBUT was evaluated as being equivalent to the time the eye was kept open. Using the recorded interferometry videos, measurements were taken twice, and the averaged value was used as the measurement value.

### 2.5. Evaluation of HOAs Using VK

**Figure 1:**
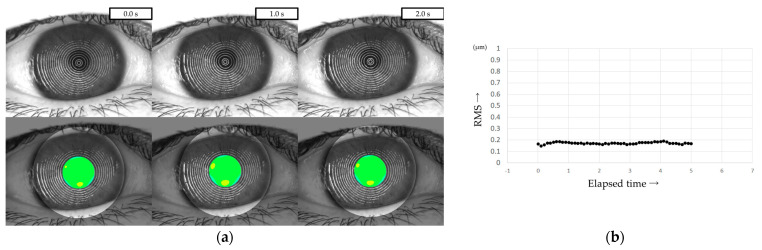
Measurement of HOAs using VK. (**a**) Upper column: MR images obtained via VK. Lower column: HOAs calculated from MR images. MR images were analyzed with a Zernike polynomial, and the HOAs were described as the RMS (µm) of the wavefront aberrations at a 4-mm pupil diameter. (**b**) HOAs evaluated every 0.1 s were automatically graphed, allowing for the evaluation of changes over time.

All patients were instructed to keep their eyes open for at least 5 s, and MR images were captured on video using a VK (RET-700; Rexxam Co., Ltd., Osaka, Japan) at 10 frames/second. The obtained MR image data was used for calculating corneal curvature, and aberrations between the ideal wavefront and that obtained from the calculated corneal curvature were analyzed with a Zernike polynomial using custom-made analysis software. HOAs were then calculated as the root mean square (RMS, in µm) of the wavefront deviation at a 4-mm pupil diameter (Figure 1). This custom-made analysis software is incorporated into the software used in our previous study of DED evaluation using VK [32].

HOAs were evaluated every 0.1 s from immediately after eye opening (0 s) to 5 s after eye opening. For frames in which the HOAs were difficult to measure due to momentary eye movements, the same values as in the frames immediately before or after were used, considering the sequential dynamics of the TF. The HOAs at the time points immediately after eye opening (i.e., 0 s), 1 s, 2 s, 3 s, 4 s, and 5 s after eye opening were defined as HOAs(0), HOAs(1), HOAs(2), HOAs(3), HOAs(4), and HOAs(5), respectively.

### 2.6. Evaluation of FBUT and CED

The patients’ eyes were stained with fluorescein strips (Ayumi Pharmaceutical Corporation, Tokyo, Japan) for FBUT measurement and CED score evaluation. FBUT was measured as the time after eye opening until pre-corneal fluorescein breakup was observed (i.e., an average of 3 measurements). Finally, CED was assessed based on the National Eye Institute staining-score grading system, and the severity of CED in the central region of the cornea was evaluated as the CED score (grades 0–3, with 0 being the best) [37].

### 2.7. Environmental Conditions

In order to avoid an increase in aqueous tear volume caused by eye drops, all examinations were performed after ensuring that the patient had not used any eye drops at least for 1 h prior to undergoing the examination. In addition, all examinations were performed in the morning (between 9:00 a.m. and 12:00 noon) at an average temperature of 24.3 ± 0.57 °C and an average humidity of 51.0 ± 4.76%.

### 2.8. Statistical Analysis

First, to investigate changes in HOAs after eye opening, HOAs at 6 time points (i.e., HOAs(0), HOAs(1), HOAs(2), HOAs(3), HOAs(4), and HOAs(5)) were compared using the Tukey-Kramer test. Next, correlations between HOAs at 6 time points and subjective/objective parameters (i.e., the summary score of DEQS, TMR, IG, SG, NIBUT, FBUT, and CED score) were evaluated in a univariate analysis. The Pearson correlation coefficient was adopted for the analysis of correlations with the TMR, NIBUT, FBUT, and DEQS summary scores as continuous variables, and the Spearman correlation coefficient was adopted for the analysis of correlations with the IG, SG, and CED scores as ordinal variables. Finally, HOA parameters that were deemed highly correlated with other parameters were selected based on the results of the univariate analysis, and the factors determining them were analyzed by multiple regression analysis in a stepwise regression. Statistical analysis was performed using JMP version 13.0 software (SAS Institute Inc., Cary, NC, USA) and Microsoft Windows Operating System (Microsoft Corporation, Redmond, WA, USA), and a *p*-value of < 0.05 was considered statistically significant.

## 3. Results

### 3.1. Subjective and Objective Parameters

In the 96 cases, the summary score of DEQS was 59.5 ± 23.1, TMR was 0.190 ± 0.097 mm, IG was 2.96 ± 1.20, SG was 2.07 ± 1.24, NIBUT was 3.68 ± 3.16 s, FBUT was 2.63 ± 2.17 s, and the CED score was 0.78 ± 1.18 (all mean ± SD).

### 3.2. Continuous Measurement of HOAs with VK

**Figure 2:**
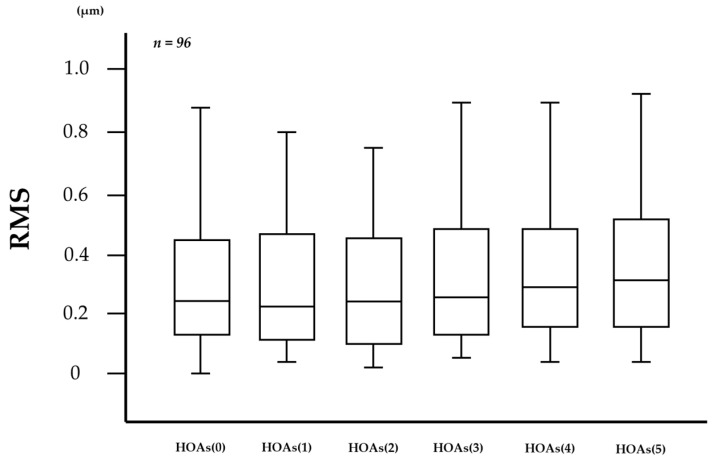
Changes in HOAs over time after eye opening. HOAs appeared to be stable for 5 s after eye opening, with no significant differences in the comparison of any two HOA parameters (all *p* > 0.130, Tukey-Kramer test).

In the measurement of HOAs every 0.1 s via VK, the number of frames in which HOAs were unmeasurable was 86 (1.79%) out of 4800 frames in the 96 eyes. As shown in Figure 2, 1-s evaluations of HOAs showed that HOAs(0), HOAs(1), HOAs(2), HOAs(3), HOAs(4), and HOAs(5) were 0.503 ± 1.133, 0.357 ± 0.389, 0.310 ± 0.262, 0.414 ± 0.693, 0.375 ± 0.410, and 0.685 ± 2.094 µm (mean ± standard deviation), respectively, and were unchanged without significant differences in the comparison of any two HOA parameters (all *p* > 0.130), including the comparison between HOAs(0) and HOAs(5) (*p* = 0.833).

### 3.3. Relationship between HOAs and Other Parameters

As shown in Table 1, the respective correlations between the HOAs and the TMR, IG, SG, NIBUT, FBUT, CED score, and summary score of DEQS were as follows: r = −0.203 (*p* = 0.047), 0.434 (*p* < 0.001), 0.348 (*p* < 0.001), −0.292 (*p* = 0.004), −0.284 (*p* = 0.005), 0.503 (*p* < 0.001), −0.136 (*p* = 0.187) for HOAs(0); r = −0.141 (*p* = 0.170), 0.439 (*p* < 0.001), 0.431 (*p* < 0.001), −0.414 (*p* < 0.001), −0.345 (*p* < 0.001), 0.536 (*p* < 0.001), −0.052 (*p* = 0.614) for the HOAs(1); r = −0.146 (*p* = 0.157), 0.378 (*p* < 0.001), 0.419 (*p* < 0.001), −0.417 (*p* < 0.001), −0.386 (*p* < 0.001), 0.517 (*p* < 0.001), −0.167 (*p* = 0.103) for the HOAs(2); r = −0.141 (*p* = 0.172), 0.387 (*p* < 0.001), 0.435 (*p* < 0.001), −0.311 (*p* = 0.002), −0.313 (*p* = 0.002), 0.537 (*p* < 0.001), −0.075 (*p* = 0.469) for HOAs(3); r = −0.162 (*p* = 0.116), 0.364 (*p* < 0.001), 0.413 (*p* < 0.001), −0.379 (*p* < 0.001), −0.370 (*p* < 0.001), 0.523 (*p* < 0.001), −0.021 (*p* = 0.839) for the HOAs(4); and r = −0.050 (*p* = 0.626), 0.361 (*p* < 0.001), 0.391 (*p* < 0.001), −0.161 (*p* = 0.117), −0.175 (*p* = 0.089), 0.500 (*p* < 0.001), −0.158 (*p* = 0.124) for the HOAs(5).

### 3.4. Factors Determining HOAs

Based on the results of the univariate analysis (refer to Section 3.2, above), HOAs(1), HOAs(2), HOAs(3), and HOAs(4) were correlated with multiple ocular surface abnormality parameters (with the exception of TMR and the summery score of DEQS). Of these, HOAs(1) and HOAs(2), which have a short elapsed time from eye opening, were selected for multiple regression analysis. As shown in Table 2, multiple regression analysis revealed that both HOA parameters were determined by the NIBUT and CED scores, each expressed as follows:

HOAs(1) = 0.347 + (−0.028 × NIBUT) + (0.144 × CED score) (R2 = 0.606, *p* < 0.001).

HOAs(2) = 0.323 + (−0.021 × NIBUT) + (0.084 × CED score) (R2 = 0.625, *p* < 0.001).

### 3.5. Representative Cases

**Figure 3:**
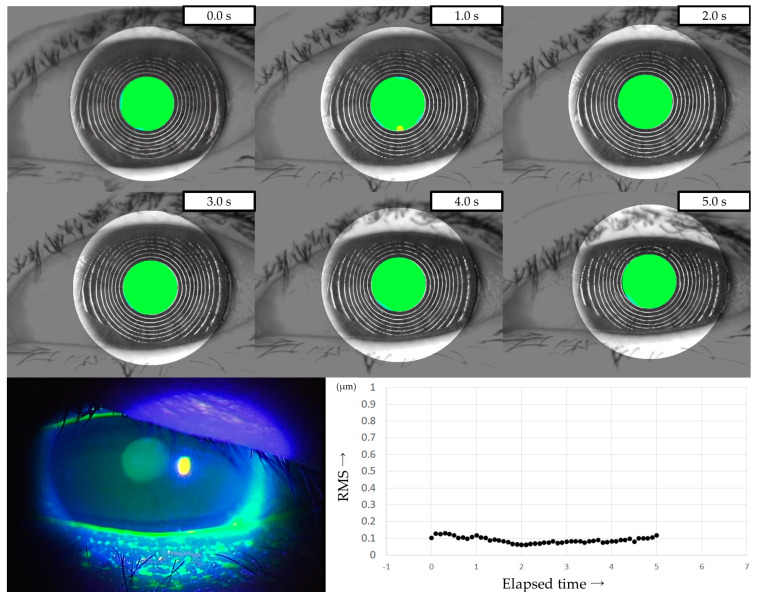
Case of a 49-year-old female. Stable TF and sufficient aqueous tear volume without CED are presented in this case; HOAs were small and stable, corresponding to IEDE based on the classification for DED via breakup pattern [36].

**Figure 4:**
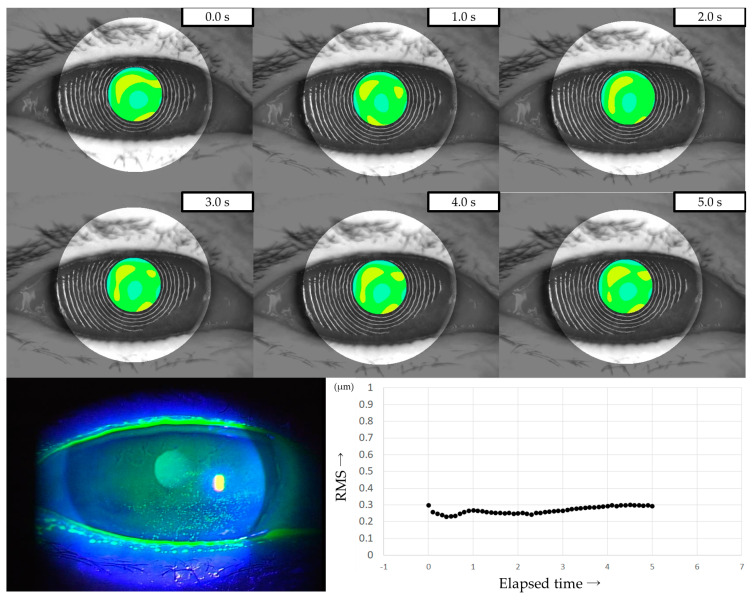
Case of a 79-year-old female. Decreased aqueous tear volume, mild TF instability, and CED are presented in this case. Increased HOAs were observed immediately after eye opening, corresponding to ADDE based on the classification for DED via breakup pattern [36].

**Figure 5:**
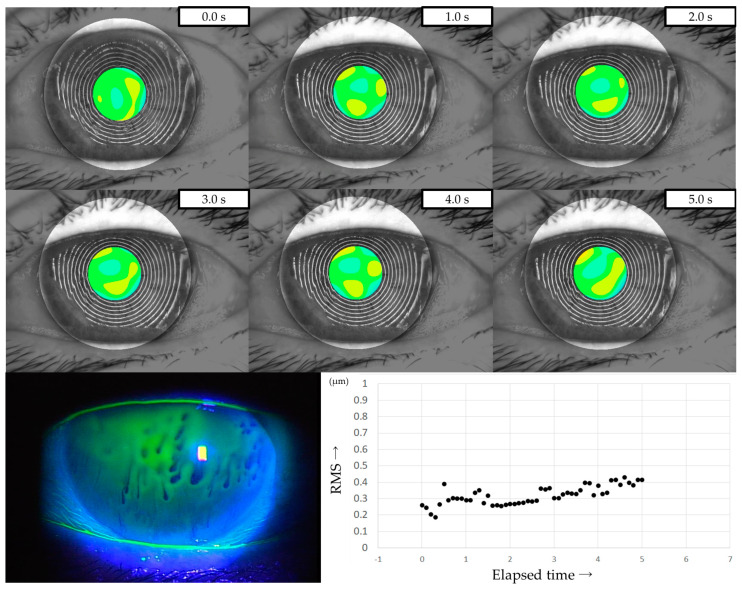
Case of a 49-year-old female. Stable TF and sufficient aqueous tear volume without CED are presented in this case; HOAs were small and stable, corresponding to DWDE based on the classification for DED via breakup pattern [36].

Three representative cases are presented below. The detailed clinical features of these three cases are shown in Table 3. Case 1 was the left eye of a 49-year-old female (Figure 3). TMR was 0.273 mm, IG and SG were 4 and 1, respectively, NIBUT and FBUT were 8.31 and 4 s, respectively, and the CED score was 0. HOAs were stable and small throughout the measurement time. Case 2 was the right eye of a 79-year-old female (Figure 4). TMR was 0.095 mm, IG and SG were both 2, NIBUT and FBUT were 3.32 and 2 s, respectively, and the CED score was 1. HOAs were observed immediately after eye opening and were higher than in Case 1. Case 3 was the left eye of a 77-year-old female (Figure 5). TMR was 0.245 mm, IG and SG were 2 and 1, respectively, NIBUT and FBUT were both 0 s, and the CED score was 0. HOAs were observed immediately after the eye opening and were comparable to those in Case 2.

## 4. Discussion

HOAs are complex refractive errors that occur when the wavefront of light passes through the eye with irregular refractive components, including the TF, the cornea, and the lens. Generally, HOAs are unlikely to cause significant visual loss, but they can cause image defocus and distortion and cannot be corrected with glasses or contact lenses [38]. HOAs are observed in normal eyes and are reported to increase after eye opening [20,39], and there are several patterns of HOAs after the eye is kept open [40]. Meanwhile, HOAs in DED patients show increased HOAs compared with normal eyes [15,22]. In eyes with DE, factors such as decreased aqueous tear volume, decreased wettability of the cornea, and increased evaporation from TF are thought to decrease TF stability and may result in CED [41], and these factors may contribute to increased HOAs.

Reportedly, superficial punctate keratopathy (SPK) in the central area of the cornea, i.e., the pupillary area, can affect the optical quality of the eye. In DED cases, SPK in the pupillary area is reportedly associated with increased total HOAs and backward light scattering [17,18,22,42,43]. It is also reported that HOAs are related to TF stability, since HOAs increase after TF breakup [39]. In addition, previous methods for estimating TF breakup time based on HOAs have been reported [44]. Koh described the mechanism of degraded VF in DED as TF instability, which leads to increased forward light scattering and decreased stability of post-blink HOAs, as well as ocular surface damage, which leads to increased backward light scattering and increased baseline HOAs [42]. According to this, the evaluation of HOAs in DED would ideally be able to evaluate the effects of TF instability and CED as ocular surface damage, which are the essential factors of DED, concurrently.

In this study, we attempted to measure HOAs using VK in DED patients and investigated the ocular surface factors that determine HOAs. We found that our VK system was capable of measuring detailed HOAs every 0.1 s in DED patients, and the number of frames that were unmeasurable due to momentary eye movements (i.e., frames considered equivalent to the HOAs of the previous or next frame) were less than 2% in all cases, indicating that the quality of the examination was adequate.

Analysis of the relationship between HOA and parameters of ocular surface abnormality revealed that HOA, especially at 1~2 s after eye opening, was closely related to multiple indicators of ocular surface abnormality, as shown in Table 1. Furthermore, multivariate analysis to investigate the factors that determine them revealed that the HOAs at 1 and 2 s reflect TF instability and the degree of CED. To the best of our knowledge, this is the first report showing that these two essential factors of DED affect HOAs concurrently. First, and as in previous reports, our findings revealed an obvious association between HOAs and CED in the central area of the cornea [17,22,42]. The fact that the CED score showed a moderate or better positive correlation with HOAs at each time point after eye opening in the univariate analysis suggests that the CED affects optical quality regardless of the time after eye opening and is considered to correspond to the baseline HOAs that Koh described [42]. In addition, the multivariate analysis results shown in Table 2 suggest that CED in the central area of the cornea may have a greater impact on VF than TF instability. Second, HOAs were found to be associated with several TF stability indicators, either qualitative (IG and SG) or quantitative (NIBUT and FBUT). Furthermore, the correlations between HOAs and these indicators showed different results depending on the time point after eye opening, in contrast to the CED. This suggests that HOAs are affected by time-dependent TF dynamics (i.e., TF formation and breakup of TF) after eye opening.

Using a slit lamp with fluorescein staining is an effective method for observing TF dynamics after eye opening. A diagnostic method named Tear Film Oriented Diagnosis (TFOD) was previously developed in Japan to classify DED into three subtypes, i.e., ADDE, decreased wettability dry eye (DWDE), and increased evaporation dry eye (IEDE), by evaluating the fluorescein breakup pattern (FBUP) of a closed fluorescein-stained eye and then quickly opening the eye to detect the insufficient component in the TF [36]. TF is formed in two steps: (1) deposition of an aqueous tear on the cornea by the upper eyelid and (2) upward spreading of the TFLL [36,45,46], and approximately 1–2 s is thought to be required for these processes to form a complete TF on the whole cornea [47]. All FBUPs except for random breaks (suggestive of IEDE) appear before the establishment of TF and are thus often observed immediately or 1–2 s after eye opening. For example, line breaks suggestive of ADDE show a linear-shaped breakup in lower areas of the cornea during the formation of TF due to the decrease in aqueous tear volume. In DWDE, qualitative and quantitative abnormalities of mucin−16 of the corneal surface epithelium are thought to decrease corneal wettability [48,49], resulting in spot break, which shows as an oval-shaped breakup around the center of the cornea due to insufficient aqueous tear deposition on the cornea when the eye is opening, and dimple break, which shows as a horizontal breakup due to aborted upward spread of the TFLL after eye opening. In consideration of such TF dynamics, HOAs at 1 or 2 s after eye opening are reasonably reflecting TF instability, and the results in our study are also supportive of this. HOAs(5) showed less correlations with NIBUT and FBUT than HOAs at other time points, possibly because the timing of HOAs was less related to the formation and breakup of TF.

These findings and those of previous reports suggest that evaluation of the HOA around 1–2 s after eye opening in DED patients may be most closely correlated with the optical and anatomical abnormalities of the corneal surface within the optical zone. In this study, HOAs at every 1 s were targeted for analysis, although measurements were conducted every 0.1 s when performing VK. Although some of the measured HOAs may be more highly correlated with TF stability and CED, it would be reasonable and sufficient to ensure the quality of future studies of HOAs in DED by using simple indicators such as 1 or 2 s after eye opening. Furthermore, this method may also be beneficial in the evaluation of severe DED patients who experience pain with prolonged eye opening.

The three representative cases presented in Section 3.5 showed intriguing features. Case 1 was diagnosed as IEDE since a random break was observed as the FBUP. With sufficient aqueous tear volume and stable TF, without CED, HOAs were minimal throughout the measurement duration, indicating that degraded VF due to DED would be minimal in that case. Case 2 was diagnosed as ADDE since a line break was observed as the FBUP. Decreased tear volume, moderate CED, and mild TF instability were observed, which resulted in elevated HOA values throughout the measurement duration. This finding suggests that the patient may have had degraded VF due to TF instability and CED. Case 3 was diagnosed as DWDE since a spot break was observed as the FBUP. Despite sufficient aqueous tear volume and the absence of CED, the TF instability was severe. The resulting HOAs were comparable to those of Case 2, thus suggesting that the patient may have had degraded VF mainly due to TF instability. Those three cases suggest that TF instability and CED may affect VF with increased HOAs through different mechanisms, which would partially be supportive of the mechanism of visual dysfunction mentioned by Koh [42]. 

Although TFOD is considered a breakthrough diagnostic method, it has the disadvantage of being invasive due to the necessity of contacting the patient’s eye with a fluorescein strip and the possibility of significant differences in determining results due to varying staining techniques [50]. In particular, spot break, as found in Case 3, is generally not accompanied by CED and is considered one of the breakup patterns in short-BUT type DED [36,41,51]. Subjective symptoms of short-BUT type DED are generally considered to be severe [51], and according to a multicenter study conducted in Japan, subjective symptoms evaluated by DEQS of DWDE with spot break were reportedly comparable to those observed in severe ADDE with area break [52]. Of note, the detection of spot breaks requires both appropriate fluorescein staining without increasing aqueous tear volume and proper instruction to the patient regarding the blinking process, and even its non-invasive detection with a wavefront analyzer is reportedly difficult [53]. Thus, spot breaks can easily be missed [36]. We have previously developed a new evaluation method using VK and reported that it successfully reflects ocular surface abnormalities in DED [32] and can be applied to non-invasive detection of spot breaks through careful observation of MR images during the first 2 s after eye opening [54]. In addition, the findings in this current study indicate that VK may have the potential ability to detect and evaluate DED, especially DWDE, that can be easily missed, both in terms of ocular surface abnormalities as well as VF, i.e., both morphological and functional. 

Conversely, no correlation was found between HOAs and DEQS at any time after eye opening. Previous studies have reported that there is generally no or minimal association between objective findings and subjective symptoms in studies of DED [55,56], while Denoyer et al. have reported intriguing findings that an increased index of HOAs after eye opening correlates with subjective symptoms assessed by the Ocular Surface Disease Index [24,57]. As shown in Figure 2 in this current study, no increasing trend in HOAs was observed. Furthermore, previous studies have reported that prolonged eye opening in DED patients leads to degraded VF in daily life [58] and that DED patients with short BUT show degraded optical quality with suppressed blinking due to visual display terminal work [59]. Considering those reports, we should take not only abnormalities of TF and cornea epithelium but also blinking and the possibility of pupil diameter being smaller than 4 mm in our daily lives into consideration when degraded VF in DED is evaluated. Moreover, ocular symptoms would be reflected in not only the abnormalities at the central part of the cornea but also the abnormalities at the peripheral part of the cornea and even at the conjunctiva. Therefore, it may be difficult to properly evaluate the symptoms of DED patients in daily life through the methods used in the present study, in which measurement time and area were limited to within 5 s and within a 4 mm diameter area at the central part of the cornea. This limitation may be one of the reasons why we could not find an expectable relationship between HOAs and DEQS in this study. 

This study had several limitations. First, we did not evaluate the actual VF of the patients in this study, and it is unclear how the optical abnormalities described by the HOA in DED patients would affect their VF-related symptoms. For a detailed assessment of subjective symptoms related to the patients’ VF, it may be preferable to use another questionnaire specialized for VF, such as the ‘Visual Function Questionnaire-25’ [60], instead of the DEQS used in this study. Second, since normal eyes were not included in this study, the difference between normal eyes and DED patients in the measurement of HOAs using our VK system is unknown. However, the MR image of the central area of the cornea has been reported to be stable for a long time after eye opening in VK imaging in normal eyes [61], and we have confirmed similar results in preliminariy experiments. Furthermore, obvious differences in HOAs in normal and DE cases have previously been reported, as discussed above [17,40]. Third, while we investigated only total HOAs in this study, factors related to the components of HOAs such as coma-like aberration and spherical-like aberration are unknown. For example, increased coma-like aberration in DED suggests increased vertical asymmetry in the thickness of the TF [43]. Whether and how the irregular astigmatic component of HOAs is affected by factors related to TF stability and patterns of CED would be intriguing and would require further study. Finally, the current study excluded cases with other factors that might increase HOAs, such as various corneal diseases, ocular surface diseases other than DED, or a history of laser refractive surgery [62,63]. It would be difficult to evaluate the VF of DED cases with factors affecting such an unexpected increase in HOAs using only VK.

## 5. Conclusions

In conclusion, we successfully used VK to perform continuous HOA measurements in DED and showed that measuring HOAs at 1 or 2 s after eye opening in DED patients can be useful in estimating degraded VF caused by TF instability and CED in the central areas of the cornea. This finding may be useful for non-invasive, objective, and rapid evaluation of complex decreased VF in DED.

## Figures and Tables

**Table 1:** Correlation between HOAs at every second from 0 to 5 s after eye opening and other parameters (*n* = 96).

	HOAs(0)			HOAs(1)			HOAs(2)			HOAs(3)			HOAs(4)			HOAs(5)		
versus	r		*p*	r		*p*	r		*p*	r		*p*	r		*p*	r		*p*
TMR	−0.203	*	0.047	−0.141	*	0.170	−0.146	*	0.157	−0.141	*	0.172	−0.162	*	0.116	−0.050	*	0.626
IG	0.434	**	<0.001	0.439	**	<0.001	0.378	**	<0.001	0.387	**	<0.001	0.364	**	<0.001	0.361	**	<0.001
SG	0.348	**	<0.001	0.431	**	<0.001	0.419	**	<0.001	0.435	**	<0.001	0.413	**	<0.001	0.391	**	<0.001
NIBUT	−0.292	*	0.004	−0.414	*	<0.001	−0.417	*	<0.001	−0.311	*	0.002	−0.379	*	<0.001	−0.161	*	0.117
FBUT	−0.284	*	0.005	−0.345	*	<0.001	−0.386	*	<0.001	−0.313	*	0.002	−0.370	*	<0.001	−0.175	*	0.089
CED score	0.503	**	<0.001	0.536	**	<0.001	0.517	**	<0.001	0.537	**	<0.001	0.523	**	<0.001	0.500	**	<0.001
summary score of DEQS	−0.136	*	0.187	−0.052	*	0.614	−0.167	*	0.103	−0.075	*	0.469	−0.021	*	0.839	−0.158	*	0.124

HOAs: higher-order aberrations; TMR: tear meniscus radius; IG: tear film lipid layer interference grade; SG: tear film lipid layer spread grade; NIBUT: non-invasive breakup time; FBUT: fluorescein breakup time; CED: corneal epithelial damage; DEQS: Dry Eye Related Quality of Life Score. * Pearson’s correlation coefficients; ** Spearman’s correlation coefficient; *p* < 0.05 was considered statistically significant.

**Table 2:** HOAs(1) and HOAs(2) multiple regression analysis results.

**HOAs(1)**	**RC**	**SRC**	**SE**	***t*-Value**	***p*-Value**	
Intercept	0.347	0	0.066	5.25	<0.001	*
NIBUT	−0.028	−0.227	0.012	−2.41	0.018	*
CED score	0.144	0.436	0.031	4.62	<0.001	*
**HOAs(2)**	**RC**	**SRC**	**SE**	***t*-Value**	***p*-Value**	
Intercept	0.323	0	0.046	7.05	<0.001	*
NIBUT	−0.021	−0.255	0.008	−2.64	0.010	*
CED score	0.084	0.376	0.021	3.89	<0.001	*

HOAs(1) was found to be determined by NIBUT and CED score and was described by the equation: 0.347 + (−0.028 × NIBUT) + (0.144 × CED score) (R2 = 0.606, *p* < 0.001), and HOAs(2) was also found to be determined by NIBUT and CED score and was described by the equation: 0.323 + (−0.021 × NIBUT) + (0.084 × CEDS) (R2 = 0.625, *p* < 0.001). * *p* < 0.05 was considered statistically significant. HOAs: higher-order aberrations; RC: regression coefficient; SRC: standard regression coefficient; SE: standard error; NIBUT: non-invasive breakup time; CED: corneal epithelial damage score.

**Table 3:** Subjective and objective parameters of representative cases.

	Case 1	Case 2	Case 3
Age, Sex	47, Female	79, Female	77, Female
FBUP	Random break	Line break	Spot break
Subtypes of DE	IEDE	ADDE	DWDE
Summary score of DEQS	91.7	26.7	20.0
TMR (mm)	0.273	0.095	0.245
IG	4	2	2
SG	1	2	1
NIBUT (s)	8.31	3.32	0
FBUT (s)	4	2	0
CED score	0	1	0
HOAs(0) (RMS, µm)	0.104	0.546	0.408
HOAs(1) (RMS, µm)	0.110	0.430	0.696
HOAs(2) (RMS, µm)	0.064	0.473	0.765
HOAs(3) (RMS, µm)	0.076	0.420	0.701
HOAs(4) (RMS, µm)	0.078	0.428	0.573
HOAs(5) (RMS, µm)	0.107	0.408	0.791

FBUP: fluorescein breakup pattern; DE: dry eye; IEDE: increased evaporation dry eye; ADDE: aqueous deficient dry eye; DWDE: decreased wettability dry eye; DEQS: Dry-Eye-Related Quality of Life Score; TMR: tear meniscus radius; IG: tear film lipid layer interference grade; SG: tear film lipid layer spread grade; NIBUT: non-invasive breakup time; FBUT: fluorescein breakup time; CED: corneal epithelial damage; HOAs: higher-order aberrations; RMS: root mean square.

## Data Availability

The data that support the findings of this study are available from the corresponding author upon reasonable request.

## Abbreviations

ADDE: aqueous deficient dry eye; BUT: breakup time; CED: corneal epithelial damage; DED: dry eye disease; DEQS: Dry-Eye-Related Quality of Life Score; DWDE: decreased wettability dry eye; FBUP: fluorescein breakup pattern; FBUT: fluorescein breakup time; HOAs: higher-order aberrations; IEDE: increased evaporation dry eye; IG: tear film lipid layer interference grade; MR: Meyer ring; NIBUT: non-invasive breakup time; RMS: root mean square; SG: tear film lipid layer spread grade; TF: tear film; TFLL: tear film lipid layer; TFOD: Tear Film Oriented Diagnosis; TMR: tear meniscus radius; SPK: superficial punctate keratopathy; VF: visual function; VK: videokeratographer.
